# Moroccan parents caring for children with juvenile idiopathic arthritis: positive and negative aspects of their experiences

**DOI:** 10.1186/1546-0096-11-39

**Published:** 2013-10-20

**Authors:** Nada Mawani, Bouchra Amine, Samira Rostom, Dalal El Badri, Majda Ezzahri, Fanata Moussa, Siham Shyen, Sanae Gueddari, Moudjibou Wabi, Bouchra Shkirat, Najia Hajjaj Hassouni

**Affiliations:** 1 LIRPOS, URAC30, Mohammed V. Souissi University, Rabat, Morocco; 2 Department of Rheumatology, El Ayachi hospital, University Hospital of Rabat-Sale, PO Box: 10000, Salé, Morocco; 3 Laboratory of Biostatistics, Clinical Research and Epidemiology (LBRCE), Faculty of Medicine and Pharmacy, Rabat, Morocco; 4 Department of Pediatrics Children Hospital, University Hospital of Rabat, Rabat, Morocco

**Keywords:** Juvenile idiopathic Arthritis, Caregiver, CRA, Burden, Care at home

## Abstract

**Background:**

Juvenile idiopathic arthritis (JIA) can lead to serious disability in children and adolescents, requiring intensive home care usually provided by parents .These parents must also cope with physical, familial, social and financial constraints.

The aim of this study is to evaluate the positive and negative impacts of caregiving on parents to children with JIA, and identify diseases-related variables that affect these outcomes.

**Methods:**

Cross-sectional study including 47 patients diagnosed with JIA defined by the International League of association for Rheumatology (ILAR) 2001 classification. Socio-demographic, clinical and biological data related to patient and disease were collected. Positive and negative effects of caregiving on parents of children with JIA were assessed via a validated instrument; the Caregiver Reaction Assessment (CRA).The CRA assesses parent’s self-esteem, financial problems, health problems, disrupted schedule and lack of family support. All parents completed the CRA questionnaire. A statistical analysis was conducted to determine the influence of disease-related variables on caregivers.

**Results:**

Forty-seven patients were included with 40.4% female. The average patient age was 11 years, and a mean patient body mass index (BMI) was 18. Forty patients were in school. Median disease duration of JIA was 4 years. The most frequent arthritis subtype was persistent oligoarthritis in 12-patients. Nearly 15% had extra-articular manifestations most frequently ocular involvement (6.4%). Median of global Visual analogic scale (VAS) was 20 and median Child health assessment questionnaire (CHAQ) was 0. The primary caregiver was the mother for all patients. Mean maternal age was 38 years, 42% of mothers were illiterate, and nearly all (95%) were without employment. The mean values of different dimensions of the CRA were respectively: self-esteem 3.5, financial problems 3.7, health problem 2.4, disrupted schedule 3.6 and familial support 2.9. Disrupted schedule of parents was correlated with disease severity assessed by physician VAS (p = 0.02). Financial problems of parents were significantly associated with disease duration (p = 0.04). There was no significant association between the type of JIA, activity or severity of the disease and other dimensions of the CRA.

**Conclusion:**

This study suggests that the management of children with JIA has a high negative impact among caregiving parents, represented mainly by the disruption of their activities, the lack of family support, financial problems and health problems. However, caregiving often also improves caregiver’s self-esteem (feeling of gratification to be helping).

## Background

Juvenile idiopathic arthritis (JIA) is the most common chronic inflammatory arthritis in children, and has dramatic consequences. It encompasses a heterogeneous group of disease including inflammatory joint with no apparent etiology. It is the fifth chronic disease in children, with an incidence that ranges between 2 and 20/100000. Prevalence rates ranging between 16 and 150/ 100,000 have been reported [1]. In Morocco, there is no available national prevalence of JIA.

Children with JIA could report a limitation of their activities because of pain, stiffness, and fatigue, which causes an impact on their autonomy and quality of life.Most home-based assistance to children with JIA, is provided by relatives and not by professional caregivers [2]. The role of caregiver comprises the provision of the care itself, as well as provision of the expenses necessary for access to professional help, or for the acquisition of special equipment. This imposes a significant burden on caregivers. Caregivers can experience alterations in their psychological and physical health [3]. They often indicate that their care tasks are demanding, especially when they have to perform unpleasant tasks, feel they have no time left for themselves, or when they become socially isolated. As a result, caregivers of patients with chronic diseases including oncology, may feel strained, become depressed, or develop health problems [4].

Then parents of children with JIA have to adapt their lifestyle to the requirements of their children's disease, including medical monitoring, treatment and disease progression. Thus the World Health Organization has defined the needs of the family and caregivers as a priority in palliative care [2]. Provision of support to caregivers is an essential part of community care, and defining caregiver’s burden is an important part of this target.

In Morocco, the access to care is limited. Most patients cannot access home-based professional care. Indeed, 15% of the population has medical cover, 60% of healthcare costs are paid by households and to improve access to care for rural population and destitute, the Medical Assistance Plan based on the principles of social assistance and national solidarity in favor of the poor was created in 2002 [5]. Nevertheless, more than three million Moroccans suffer from chronic diseases, which require relatively expensive treatments. The repayment rate ranges from 70% to 100% for patients with medical cover.

On the other hand, there is a strong family engagement, which means that family provides the majority of patient care at home. In fact the family institution is particularly important in Moroccan society [6]. Family links in most instances are beyond the strict framework of the conjugal couple to include parents and children, ascendants uncles, aunts, cousins and their descendants. This specificity attributed to the Moroccan family a dual function: the protection of the ascendants and future descendants. Therefore, this dual role is the source of the duty of care and protection of the weak, which weighs on all members of the kinship network.

The aim of this study was to evaluate the effects of caregiving among parents of Moroccan children with JIA, and to identify disease-related variables that influence these parents’ experience.

### Patients and methods

#### Study population

A cross-sectional study was conducted in JIA patients less than 16 years of age who were recruited during a hospital stay or outpatient consultation in the El Ayachi University Hospital and Children’s Hospital of Rabat. These two institutions are referral centers for all the public regional medical centers of the country. Forty-seven patients were recruited as they came into clinic. The diagnosis of juvenile idiopathic arthritis was defined according to the International league of Association for Rheumatology (ILAR) 2001 classification. Informed consent was obtained from all subjects.

## Methods

### Data collection and measurements

The demographic, clinical, and biological data related to patients were collected. These included the type of JIA, the disease duration, and the disease activity score DAS28 ESR and its different components [DAS28 ESR = 0.56 √TJC + 0.28 √SJC + 0.70* (ESR) + 0.14*PtGA) with high activity (>5.1), moderate activity (3.2 – 5.1), low activity ( 2.6- 3.2) and remission (<2.6)], functional impairment assessed by Moroccan validated version of the Child health assessment questionnaire (CHAQ) with score of 0–3 (3 = worst functioning) [7], and characteristics of parents were recorded.

### Assessment of positive and negative dimension of the experience of parents

The subjective burden of informal care refers the informal caregiver’s perception of the impact of the objective burden related to caregiving.

We assessed the subjective burden of caregiving for parents of children with JIA with the Caregiver Reaction Assessment (CRA) instrument [2]. The CRA was developed and validated in the USA among caregivers of persons with all types of chronic physical and mental impairments [4,8], the Dutch version was found to be valid among caregivers of colorectal cancer patients [8], and the French form was validated in partners of chronic disease patients [2].

The French version of the CRA was translated into Arabic dialect by two accredited translators, then against-translated by two other accredited translators, and all parents of patients filled the final version, assisted by the same physician for those illiterate.

The CRA consists of 24 items on five dimensions that assess both the positive burden of caregiving ('care-derived self-esteem’) and the negative burden of caregiving ('lack of family support’, 'financial problems’, 'disrupted schedule’ and 'loss of physical strength’), as described in Table 1. Each item is rated on a five-point scale, ranging from 'strongly disagree’ to 'strongly agree’. For each dimension, a total score was computed as the average of the item scores, ranging from 1.0 to 5.0, and that more the score is high more the impact is important, whether it be in deleterious or good sense. A score of 1.0 represents 'no derived self-esteem’ on the positive dimension, and 'no burden’ on the negative dimensions. Unfortunately, the CRA does not have a total score that combines the five dimensions.

**Table 1:** Dimensions of the Caregiver Reaction Assessment (CRA)

**Dimension**	**Type**	**Number of items**	**Assessment**
Self-esteem	Positive	7	The extent to which care giving imparts individual self-esteem.
Financial problems	Negative	3	The adequacy, difficulty and strain of the financial situation for the Care giver and the family.
Health problems	Negative	4	The caregiver’s physical capability and energy to provide care.
Disrupted schedule	Negative	5	The extent to which care giving interrupts usual activities, causes the elimination of some activities and interferes with relaxation time.
Lack of family support	Negative	5	The extent to which the family supports and works together with the caregiver.

### Statistical analysis

Statistical analysis was performed using the SPSS Statistical package 18.0 for Windows (SPSS 18, Chicago, IL, USA). Descriptive data are expressed as mean ± SD or as percentage. The Spearman test and p-values were used to determine the strength of the association between the variables. A p value < 0.05 was considered to indicate statistical significance.

## Results

### Patients characteristics

Demographic characteristics of the study population are described in Table 2. Of a total of 47 children with JIA, 10 had a systemic arthritis, 12 persistent oligoarthritis, 2 extented oligoarthritis, 8 polyarthritis and 15 with other types of arthritis (twelve enthesitis and four undetermined arthritis). The mean age of patients was 11.5 ± 3.3, and 15% were not in school. Disease duration of JIA had a median of 4 years (range 2–6). The median of global VAS and CHAQ were respectively 20 (range10-30) and 0 (range 0–1).The DAS28 had a mean of 3.1 ± 1.5. Almost 15% of patients had an extra-articular manifestation of JIA; the most common was ocular involvement (6.4%).

**Table 2:** Characteristics of the study population

**Characteristics**	**Population (N = 47)**
Age^1^ (years)	11.5 ± 3.3
Sex(F)^2^	19 (40.4)
BMI(kg/m^2^)^1^	18.2 ± 3.9
In School^2^	40 (85.1)
Type of JIA^2^ =	
Systemic arthritis	10 (22.2)
Seropositive polyarthritis	02 (4.4)
Seronegative polyarthritis	06 (13.3)
Persistent oligoarthritis	12 (26.7)
Extented oligoarthritis	02 (4.4)
Enthesitis	11 (24.4)
Psoriatic arthritis	0
Undetermined	04 (11.7)
Disease duration (years)^3^	4 [2–6]
Global VAS^3^	20 [10–30]
DAS28	3.3 ± 1.5
CHAQ^3^	0 [0–1]

^1^mean ± standard deviation; ^2^number and percentage; ^3^median and quartiles; VAS = visual analogic scale; DAS = disease activity scale; CHAQ = Child health assessment questionnaire.

### Characteristics of the parents of JIA patients

The primary caregiver was the patient’s mother in 100%. The mean age of the mothers had a mean age of 38.5 ± 8.5 years; the mothers were illiterate 42% of the time and most were employed (91.5%) (Table 3).

**Table 3:** Main characteristics of the parents of JIA patients

**Characteristics**	**Parents of JIA patients (N = 47)**
Sex (F)^1^	47 (100)
Age ^2^ (years)	38.5 ± 8.5
Employment status^1^	
Housekeeping	43 (91.5)
Employed	04 (8.5)
Educational level^1^	
Analphabet	19 (42.2)
Primary	19 (42.2)
Secondary	06 (13.3)
University	01 (2.2)

^1^number and percentage; ^2^mean ± standard deviation.

### Subjective burden results

The positive dimension of caregiver burden and self-esteem had a mean score of 3.5 ± 0.4. The mean scores on the negative burden dimensions were 3.6 ± 0.7 for disrupted schedule, 2.9 ± 0.6 for lack of family support, 3.7 ± 0.5 for financial problems, and 2.4 ± 0.6 for the loss of physical strength (Table 4).

**Table 4:** Subjective caregiver burden (CRA) among partners of JIA patients

**Dimension**	***Mean ± standard deviation***
Self-esteem	3.5 ± 0.4
Financial problems	3.7 ± 0.5
Loss of physical strength	2.4 ± 0.6
Disrupted schedule	3.6 ± 0.7
Lack of family support	2.9 ± 0.6

### Correlation between subjective caregiver burden dimensions and clinical disease characteristics

The relationship between the CRA domains and clinical disease parameters were analyzed (Table 5). Disrupted schedule of parents was correlated with disease severity assessed by physician VAS (p = 0.02), there was also a statistically significant association between the duration of disease progression and financial problems of parents. However, there was no significant association between the type of JIA, activity or severity of the disease (assessed by physician VAS, the parameters of inflammation, strong positive serology and functional impact by the CHAQ) and other dimensions of the CRA.

**Table 5:** Correlation between subjective caregiver burden dimensions and clinical disease characteristics

	**Disease duration**		**Global physician VAS**		**CHAQ**	
	**r**	**p**	**r**	**p**	**r**	**p**
Self esteem	**-**0.2	0.1	0.03	0.8	0.1	0.4
Financial problems	0.2	*0.04*	0.1	0.2	**-**0.1	0.2
Loss of physical strength	**-**0.1	0.1	**-**0.2	0.1	0.09	0.5
Disrupted schedule	0.1	0.2	0.3	*0.02*	**-**0.1	0.4
Lack of family support	0.4	0.7	0.08	0.5	**-**0.08	0.5

CHAQ: Child Health Assessment Questionnaire, VAS: visual analogic scale, r: r de spearman.

### Correlation between subjective caregiver burden dimensions and biological disease characteristics

We have denoted the association of the five CRA dimensions with biological disease parameters in Table 6. Lack of family support was significantly associated with the presence of rheumatoid factor (RF) (p < 0.05). There were no significant influences between the CRA domains and the presence of anti-citrullinated protein antibody (ACPA) or inflammation parameters.

**Table 6:** Correlation between subjective caregiver burden dimensions and biological disease characteristics

	**ESR**		**CRP**		**RF +**		**ACPA +**	
	**r**	**p**	**r**	**p**	**r**	**p**	**r**	**p**
Self esteem	0.03	0.8	**-**0.1	0.5	**-**0.1	0.2	**-**0.1	0.3
Financial problems	**-**0.2	0.2	**-**0.07	0.7	**-**0.1	0.2	**-**0.01	0.9
Loss of physical strength	0.06	0.7	**-**0.2	0.2	**-**0.06	0.6	**-**0.08	0.5
Disrupted schedule	**-**0.09	0.5	**-**0.1	0.4	**-**0.2	0.1	0.04	0.7
Lack of family support	**-**0.03	0.8	**-**0.1	0.4	**-**0.3	0.02	**-**0.07	0.6

ESR = erythrocyte sedimentation rate; CRP = c reactive protein; RF = rheumatoid factor; ACPA = citrulline protein antibodies.

## Discussion

Our study shows that caregiver burden is common among parents of children with JIA in Morocco. Primary caregivers in our study were exclusively mothers, as was found by Iwamoto et al., and in other studies. Indeed, mothers of children with JIA are primarily responsible for managing most of their children’s health-related needs [9-11]. However, fathers may also be involved as well described by McNeil [12] who had examined the experience of fathers of children with juvenile arthritis.

The negative aspects of subjective caregiver burden were to a large degree caused by a financial problems and disrupted schedules, and to a smaller degree by a lack of family support and loss of physical strength (Table 4).

The relatively high levels of burden on 'financial problems’ dimension among parents of children with JIA was strongly associated with duration of disease progression (Table 5). The presence of a chronically ill child appears to generate increased family expenses. In particular, patients with longer duration of disease require more frequent visits and more use of professional and paid help. This also could be explained by the fact that only 8.5% of mothers in this study had a job and the majority of financial support comes from the father. This compares to the employment rate of woman in Morocco of 27.2% [13]. So negative experiences resulting from giving care may increase as the duration of care provision for a JIA child increases.

We found that the disruption of activities of parents was significantly associated to disease severity assessed by physician VAS (Table 5). The time spent by parents of children with JIA, especially mothers, to care for their sick children can be considerable, they often monitor their child’s pain, diet, exercise, sleep, school activities, recreational activities, and medications. Moreover, the chronic nature of JIA requires that these parents provide care for a long time and the types of caregiving tasks may change in advanced stages of the disease, where functional impairment is more important, as noted in previous studies [2,11,14-16]. Therefore, the financial burden is often added to stress related to the daily assistance and responsibility against a person losing their autonomy, as it was described by Toupin et al. [17].

The lack of family support was another predominant CRA negative dimension in our study (Table 4). Indeed, the role of caregiver can have a major impact on family as a whole, and lack of family support may produce a feeling of isolation and abandonment and resignation within the family. Overall, increased demands of caregiving and potential for low levels of support may contribute to high rates of psychological symptoms in mothers of children with JIA [10,18,19]. On the other hand, this negative aspect of subjective caregiver burden is both a perception of the caregiver and perhaps a powerful factor in preventing adequate care for the child. It seems to have external effects on the family unit, making it quite different from the other dimensions, which appear more likely to be resulting from the disease affecting the children and mothers, then the parents’ perceptions.

However, our study also shows that care of children with JIA also leads to self-esteem among mothers (Table 4). This positive aspect in providing care reflects the desire and pleasure found in caretaking. Therefore, help is the source of satisfaction among parents. Caretaking can be a source of gratification.

Our findings did not show a significant association between the type of JIA and dimensions caregiver burden as measured by the CRA in this study. However, a positive association was found in other studies [9,20]. Press et al. [20] reported that parents of children with pauciarticular JIA presented higher levels of anxiety and depression than the parents of children with polyarticular and systemic JIA. Iwamoto et al. found that caring for polyarticular JIA patients is more stressful than caring for oligoarticular JIA patients [9].

Our data must be examined bearing in mind the study limitations. It is a cross-sectional and monocentric study, which potentially causes a selection bias. Overall, our patient population reported relatively low disease activity because they had access to adequate care, which may not adequately reflect the importance of burden that would be greater when the disease activity is severe.

In our study, primary caregivers were exclusively mothers, which decrease the applicability of our findings to fathers. Finally, the burden instrument, the CRA, does not have a summary score, making it difficult to assess overall significance of the effects of disease measures on caregiver burden.

However, this study is the first in Morocco to assess the positive and negative consequences of parental assistance to children with JIA.

The results of this study can be used to develop specific intervention strategies for parents of patients with JIA in order to mitigate the negative burden of caregiving. Such interventions may strengthen the sustainable care provided at home. At first healthcare professionals must adopt effective information support to help parents better understand their children’s medical situation, especially in our society, where the rate of literacy of the female population over the age of 10 years is only 48%, and access to information has proven difficult for parents, particularly in the early stages of diseases when they were trying to come to terms with the condition, its management, and its consequences. Then development of psycho-educational interventions have an important role to play in assisting parents in then experience of life with JIA. On the other hand, creating of an organization for parents of children with JIA, including a voluntary home care and support when problems arise, exist or increase, may help to ease the situation for these parents.

## Conclusions

This study suggests that caregiver burden is common among parents of children with JIA in Morocco, and that the negative impact is mainly represented by the disruption of caregiver’s other activities, lack of family support, financial problems, and caregiver health problems.

Nevertheless, caregiving for children with JIA can also enhance self-esteem in parents who feel satisfaction and gratification in helping their children.

On the other hand, if caregiver burden contributes to adverse health outcomes, then parents may not only experience a greater burden of care, but may also have health problems themselves as a result of caregiving [2]. Parental social support has been shown to ameliorate parent distress [21], and social support programs have been shown to significantly reduce the appearance of mental health symptoms in mothers of children with JIA [21-24]. This study points out the ongoing need of these essential services for our JIA families.

## Competing interests

The authors declare that they have no competing interests.

## Authors’ contributions

NM participated in study design and drafted the manuscript. BA conceived the original idea for the study, supervised its design, performed the statistical analysis and gave critical comments on the draft manuscript. SR participated in study design and gave critical comments on the draft manuscript. BD enrolled patients, participated in data acquisition and critical revision of the manuscript. ME enrolled patients, participated in data acquisition and critical revision of the manuscript. SS enrolled patients, participated in data acquisition and critical revision of the manuscript. GS enrolled patients, participated in data acquisition and critical revision of the manuscript. FM enrolled patients, participated in data acquisition and critical revision of the manuscript. MW enrolled patients, participated in data acquisition and critical revision of the manuscript. SB enrolled patients, participated in data acquisition and critical revision of the manuscript. NHH participated in the study design, coordinated the study and gave critical comments on the draft manuscript. All authors read and approved the final manuscript.
